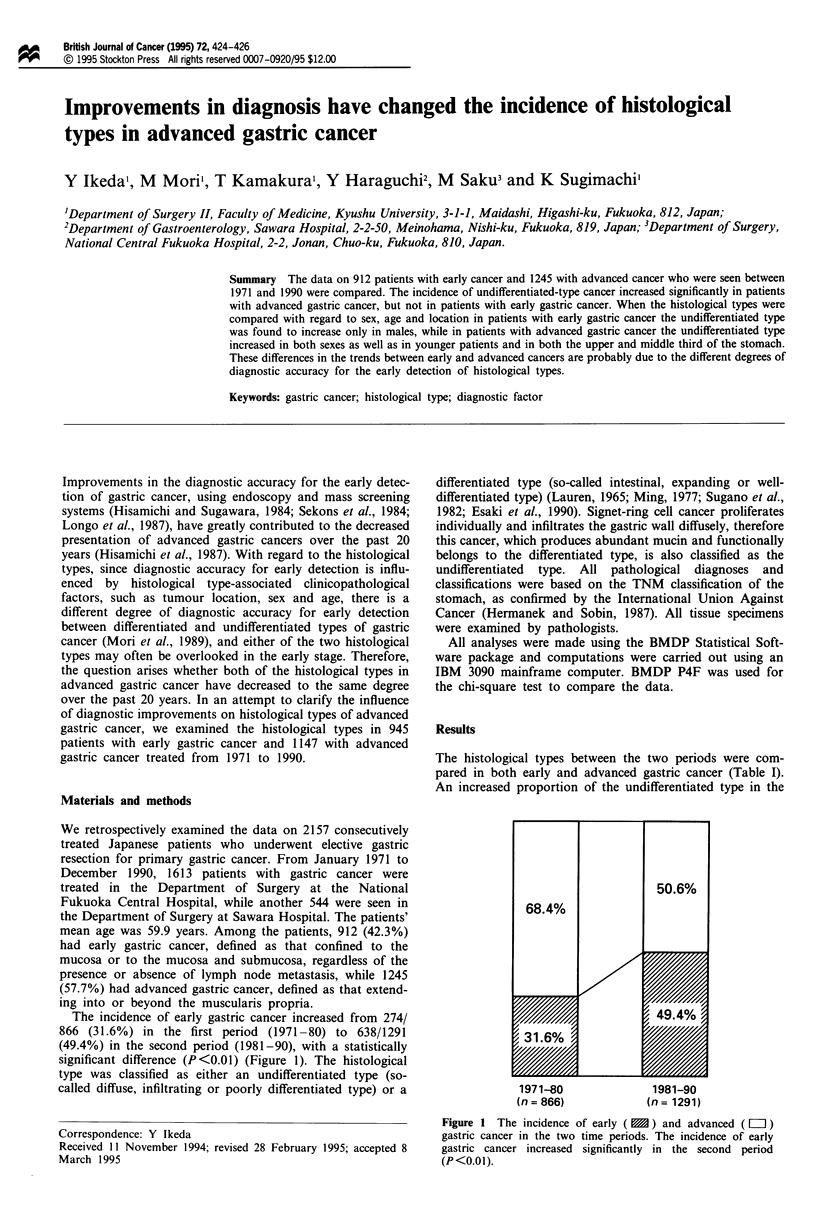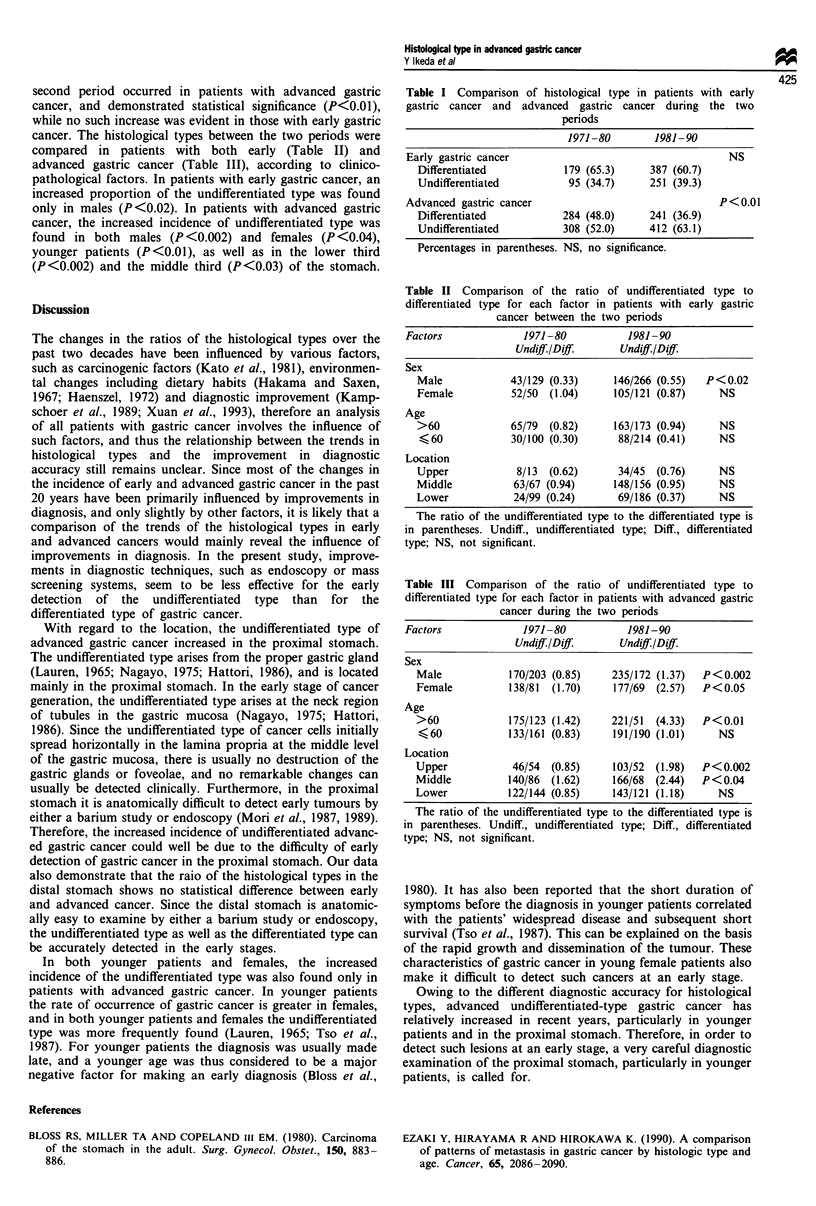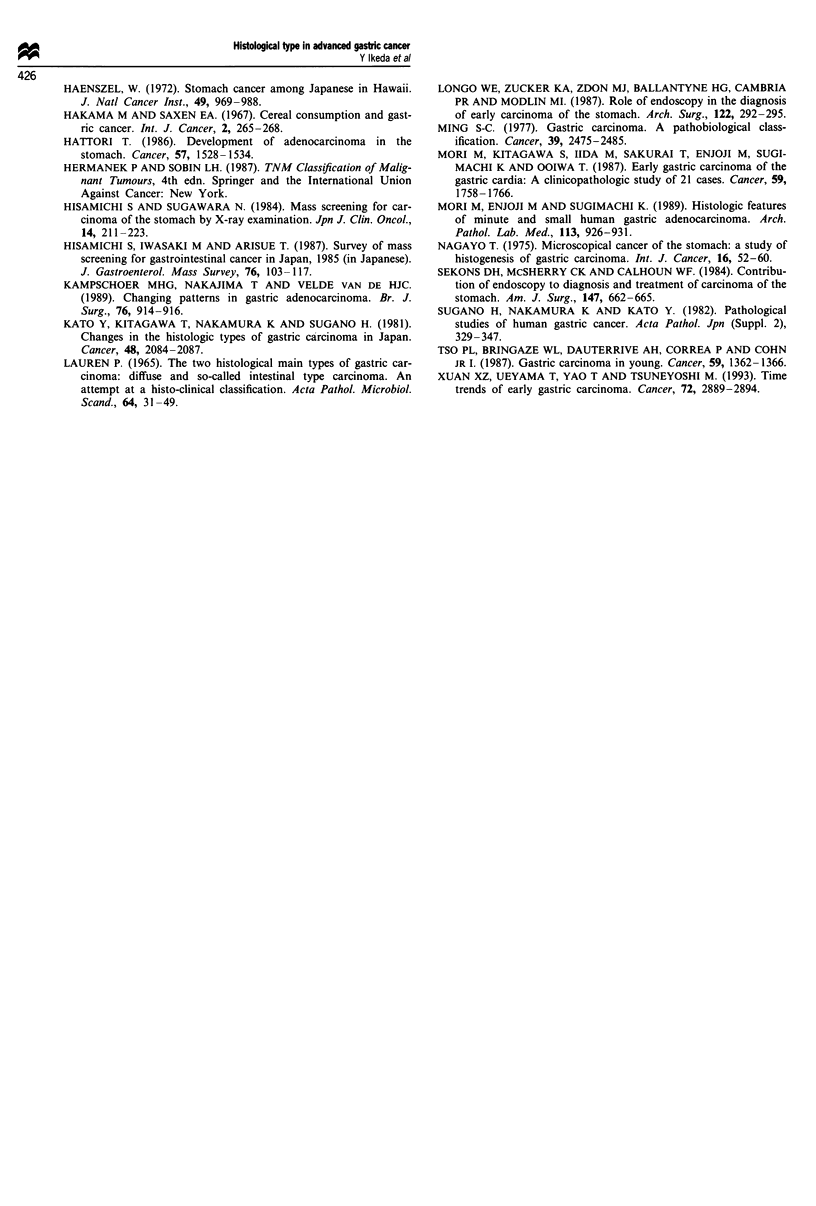# Improvements in diagnosis have changed the incidence of histological types in advanced gastric cancer.

**DOI:** 10.1038/bjc.1995.349

**Published:** 1995-08

**Authors:** Y. Ikeda, M. Mori, T. Kamakura, Y. Haraguchi, M. Saku, K. Sugimachi

**Affiliations:** Department of Surgery II, Faculty of Medicine, Kyushu University, Fukuoka, Japan.

## Abstract

The data on 912 patients with early cancer and 1245 with advanced cancer who were seen between 1971 and 1990 were compared. The incidence of undifferentiated-type cancer increased significantly in patients with advanced gastric cancer, but not in patients with early gastric cancer. When the histological types were compared with regard to sex, age and location in patients with early gastric cancer the undifferentiated type was found to increase only in males, while in patients with advanced gastric cancer the undifferentiated type increased in both sexes as well as in younger patients and in both the upper and middle third of the stomach. These differences in the trends between early and advanced cancers are probably due to the different degrees of diagnostic accuracy for the early detection of histological types.


British Journal of Cancer (1995) 72, 424–426
© 1995 Stockton Press All rights reserved 0007-0920/95 $12.00

### Improvements in diagnosis have changed the incidence of histological types in advanced gastric cancer

Y Ikeda[1], M Mori[1], T Kamakura[1], Y Haraguchi[2], M Saku[3] and K Sugimachi[1]

[1]*Department of Surgery II, Faculty of Medicine, Kyushu University, 3-1-1, Maidashi, Higashi-ku, Fukuoka, 812, Japan;* [2]*Department of Gastroenterology, Sawara Hospital, 2-2-50, Meinohama, Nishi-ku, Fukuoka, 819, Japan;* [3]*Department of Surgery, National Central Fukuoka Hospital, 2-2, Jonan, Chuo-ku, Fukuoka, 810, Japan.*

**Summary** The data on 912 patients with early cancer and 1245 with advanced cancer who were seen between 1971 and 1990 were compared. The incidence of undifferentiated-type cancer increased significantly in patients with advanced gastric cancer, but not in patients with early gastric cancer. When the histological types were compared with regard to sex, age and location in patients with early gastric cancer the undifferentiated type was found to increase only in males, while in patients with advanced gastric cancer the undifferentiated type increased in both sexes as well as in younger patients and in both the upper and middle third of the stomach. These differences in the trends between early and advanced cancers are probably due to the different degrees of diagnostic accuracy for the early detection of histological types.

**Keywords:** gastric cancer; histological type; diagnostic factor

Improvements in the diagnostic accuracy for the early detection of gastric cancer, using endoscopy and mass screening systems (Hisamichi and Sugawara, 1984; Sekons *et al.*, 1984; Longo *et al.*, 1987), have greatly contributed to the decreased presentation of advanced gastric cancers over the past 20 years (Hisamichi *et al.*, 1987). With regard to the histological types, since diagnostic accuracy for early detection is influenced by histological type-associated clinicopathological factors, such as tumour location, sex and age, there is a different degree of diagnostic accuracy for early detection between differentiated and undifferentiated types of gastric cancer (Mori *et al.*, 1989), and either of the two histological types may often be overlooked in the early stage. Therefore, the question arises whether both of the histological types in advanced gastric cancer have decreased to the same degree over the past 20 years. In an attempt to clarify the influence of diagnostic improvements on histological types of advanced gastric cancer, we examined the histological types in 945 patients with early gastric cancer and 1147 with advanced gastric cancer treated from 1971 to 1990.

### Materials and methods

We retrospectively examined the data on 2157 consecutively treated Japanese patients who underwent elective gastric resection for primary gastric cancer. From January 1971 to December 1990, 1613 patients with gastric cancer were treated in the Department of Surgery at the National Fukuoka Central Hospital, while another 544 were seen in the Department of Surgery at Sawara Hospital. The patients' mean age was 59.9 years. Among the patients, 912 (42.3%) had early gastric cancer, defined as that confined to the mucosa or to the mucosa and submucosa, regardless of the presence or absence of lymph node metastasis, while 1245 (57.7%) had advanced gastric cancer, defined as that extending into or beyond the muscularis propria.

The incidence of early gastric cancer increased from 274/866 (31.6%) in the first period (1971–80) to 638/1291 (49.4%) in the second period (1981–90), with a statistically significant difference ($P<0.01$) (Figure 1). The histological type was classified as either an undifferentiated type (so-called diffuse, infiltrating or poorly differentiated type) or a differentiated type (so-called intestinal, expanding or well-differentiated type) (Lauren, 1965; Ming, 1977; Sugano *et al.*, 1982; Esaki *et al.*, 1990). Signet-ring cell cancer proliferates individually and infiltrates the gastric wall diffusely, therefore this cancer, which produces abundant mucin and functionally belongs to the differentiated type, is also classified as the undifferentiated type. All pathological diagnoses and classifications were based on the TNM classification of the stomach, as confirmed by the International Union Against Cancer (Hermanek and Sobin, 1987). All tissue specimens were examined by pathologists.

All analyses were made using the BMDP Statistical Software package and computations were carried out using an IBM 3090 mainframe computer. BMDP P4F was used for the chi-square test to compare the data.

### Results

The histological types between the two periods were compared in both early and advanced gastric cancer (Table I). An increased proportion of the undifferentiated type in the

**Figure 1** The incidence of early (▨) and advanced (▭) gastric cancer in the two time periods. The incidence of early gastric cancer increased significantly in the second period ($P<0.01$).

Correspondence: Y Ikeda
Received 11 November 1994; revised 28 February 1995; accepted 8 March 1995

second period occurred in patients with advanced gastric cancer, and demonstrated statistical significance ($P<0.01$), while no such increase was evident in those with early gastric cancer. The histological types between the two periods were compared in patients with both early (Table II) and advanced gastric cancer (Table III), according to clinicopathological factors. In patients with early gastric cancer, an increased proportion of the undifferentiated type was found only in males ($P<0.02$). In patients with advanced gastric cancer, the increased incidence of undifferentiated type was found in both males ($P<0.002$) and females ($P<0.04$), younger patients ($P<0.01$), as well as in the lower third ($P<0.002$) and the middle third ($P<0.03$) of the stomach.

### Discussion

The changes in the ratios of the histological types over the past two decades have been influenced by various factors, such as carcinogenic factors (Kato *et al.*, 1981), environmental changes including dietary habits (Hakama and Saxen, 1967; Haenszel, 1972) and diagnostic improvement (Kampschoer *et al.*, 1989; Xuan *et al.*, 1993), therefore an analysis of all patients with gastric cancer involves the influence of such factors, and thus the relationship between the trends in histological types and the improvement in diagnostic accuracy still remains unclear. Since most of the changes in the incidence of early and advanced gastric cancer in the past 20 years have been primarily influenced by improvements in diagnosis, and only slightly by other factors, it is likely that a comparison of the trends of the histological types in early and advanced cancers would mainly reveal the influence of improvements in diagnosis. In the present study, improvements in diagnostic techniques, such as endoscopy or mass screening systems, seem to be less effective for the early detection of the undifferentiated type than for the differentiated type of gastric cancer.

With regard to the location, the undifferentiated type of advanced gastric cancer increased in the proximal stomach. The undifferentiated type arises from the proper gastric gland (Lauren, 1965; Nagayo, 1975; Hattori, 1986), and is located mainly in the proximal stomach. In the early stage of cancer generation, the undifferentiated type arises at the neck region of tubules in the gastric mucosa (Nagayo, 1975; Hattori, 1986). Since the undifferentiated type of cancer cells initially spread horizontally in the lamina propria at the middle level of the gastric mucosa, there is usually no destruction of the gastric glands or foveolae, and no remarkable changes can usually be detected clinically. Furthermore, in the proximal stomach it is anatomically difficult to detect early tumours by either a barium study or endoscopy (Mori *et al.*, 1987, 1989). Therefore, the increased incidence of undifferentiated advanced gastric cancer could well be due to the difficulty of early detection of gastric cancer in the proximal stomach. Our data also demonstrate that the raio of the histological types in the distal stomach shows no statistical difference between early and advanced cancer. Since the distal stomach is anatomically easy to examine by either a barium study or endoscopy, the undifferentiated type as well as the differentiated type can be accurately detected in the early stages.

In both younger patients and females, the increased incidence of the undifferentiated type was also found only in patients with advanced gastric cancer. In younger patients the rate of occurrence of gastric cancer is greater in females, and in both younger patients and females the undifferentiated type was more frequently found (Lauren, 1965; Tso *et al.*, 1987). For younger patients the diagnosis was usually made late, and a younger age was thus considered to be a major negative factor for making an early diagnosis (Bloss *et al.*, 1980). It has also been reported that the short duration of symptoms before the diagnosis in younger patients correlated with the patients' widespread disease and subsequent short survival (Tso *et al.*, 1987). This can be explained on the basis of the rapid growth and dissemination of the tumour. These characteristics of gastric cancer in young female patients also make it difficult to detect such cancers at an early stage.

Owing to the different diagnostic accuracy for histological types, advanced undifferentiated-type gastric cancer has relatively increased in recent years, particularly in younger patients and in the proximal stomach. Therefore, in order to detect such lesions at an early stage, a very careful diagnostic examination of the proximal stomach, particularly in younger patients, is called for.

**Table I** Comparison of histological type in patients with early gastric cancer and advanced gastric cancer during the two periods

| | *1971–80* | *1981–90* | |
|---|---|---|---|
| Early gastric cancer | | | NS |
| Differentiated | 179 (65.3) | 387 (60.7) | |
| Undifferentiated | 95 (34.7) | 251 (39.3) | |
| Advanced gastric cancer | | | $P<0.01$ |
| Differentiated | 284 (48.0) | 241 (36.9) | |
| Undifferentiated | 308 (52.0) | 412 (63.1) | |

Percentages in parentheses. NS, no significance.

**Table II** Comparison of the ratio of undifferentiated type to differentiated type for each factor in patients with early gastric cancer between the two periods

| *Factors* | *1971–80 Undiff./Diff.* | *1981–90 Undiff./Diff.* | |
|---|---|---|---|
| Sex | | | |
| Male | 43/129 (0.33) | 146/266 (0.55) | $P<0.02$ |
| Female | 52/50 (1.04) | 105/121 (0.87) | NS |
| Age | | | |
| >60 | 65/79 (0.82) | 163/173 (0.94) | NS |
| ⩽60 | 30/100 (0.30) | 88/214 (0.41) | NS |
| Location | | | |
| Upper | 8/13 (0.62) | 34/45 (0.76) | NS |
| Middle | 63/67 (0.94) | 148/156 (0.95) | NS |
| Lower | 24/99 (0.24) | 69/186 (0.37) | NS |

The ratio of the undifferentiated type to the differentiated type is in parentheses. Undiff., undifferentiated type; Diff., differentiated type; NS, not significant.

**Table III** Comparison of the ratio of undifferentiated type to differentiated type for each factor in patients with advanced gastric cancer during the two periods

| *Factors* | *1971–80 Undiff./Diff.* | *1981–90 Undiff./Diff.* | |
|---|---|---|---|
| Sex | | | |
| Male | 170/203 (0.85) | 235/172 (1.37) | $P<0.002$ |
| Female | 138/81 (1.70) | 177/69 (2.57) | $P<0.05$ |
| Age | | | |
| >60 | 175/123 (1.42) | 221/51 (4.33) | $P<0.01$ |
| ⩽60 | 133/161 (0.83) | 191/190 (1.01) | NS |
| Location | | | |
| Upper | 46/54 (0.85) | 103/52 (1.98) | $P<0.002$ |
| Middle | 140/86 (1.62) | 166/68 (2.44) | $P<0.04$ |
| Lower | 122/144 (0.85) | 143/121 (1.18) | NS |

The ratio of the undifferentiated type to the differentiated type is in parentheses. Undiff., undifferentiated type; Diff., differentiated type; NS, not significant.

### References

BLOSS RS, MILLER TA AND COPELAND III EM. (1980). Carcinoma of the stomach in the adult. *Surg. Gynecol. Obstet.*, **150**, 883–886.

EZAKI Y, HIRAYAMA R AND HIROKAWA K. (1990). A comparison of patterns of metastasis in gastric cancer by histologic type and age. *Cancer*, **65**, 2086–2090.

HAENSZEL, W. (1972). Stomach cancer among Japanese in Hawaii. *J. Natl Cancer Inst.*, **49**, 969–988.

HAKAMA M AND SAXEN EA. (1967). Cereal consumption and gastric cancer. *Int. J. Cancer*, **2**, 265–268.

HATTORI T. (1986). Development of adenocarcinoma in the stomach. *Cancer*, **57**, 1528–1534.

HERMANEK P AND SOBIN LH. (1987). *TNM Classification of Malignant Tumours*, 4th edn. Springer and the International Union Against Cancer: New York.

HISAMICHI S AND SUGAWARA N. (1984). Mass screening for carcinoma of the stomach by X-ray examination. *Jpn J. Clin. Oncol.*, **14**, 211–223.

HISAMICHI S, IWASAKI M AND ARISUE T. (1987). Survey of mass screening for gastrointestinal cancer in Japan, 1985 (in Japanese). *J. Gastroenterol. Mass Survey*, **76**, 103–117.

KAMPSCHOER MHG, NAKAJIMA T AND VELDE VAN DE HJC. (1989). Changing patterns in gastric adenocarcinoma. *Br. J. Surg.*, **76**, 914–916.

KATO Y, KITAGAWA T, NAKAMURA K AND SUGANO H. (1981). Changes in the histologic types of gastric carcinoma in Japan. *Cancer*, **48**, 2084–2087.

LAUREN P. (1965). The two histological main types of gastric carcinoma: diffuse and so-called intestinal type carcinoma. An attempt at a histo-clinical classification. *Acta Pathol. Microbiol. Scand.*, **64**, 31–49.

LONGO WE, ZUCKER KA, ZDON MJ, BALLANTYNE HG, CAMBRIA PR AND MODLIN MI. (1987). Role of endoscopy in the diagnosis of early carcinoma of the stomach. *Arch. Surg.*, **122**, 292–295.

MING S-C. (1977). Gastric carcinoma. A pathobiological classification. *Cancer*, **39**, 2475–2485.

MORI M, KITAGAWA S, IIDA M, SAKURAI T, ENJOJI M, SUGIMACHI K AND OOIWA T. (1987). Early gastric carcinoma of the gastric cardia: A clinicopathologic study of 21 cases. *Cancer*, **59**, 1758–1766.

MORI M, ENJOJI M AND SUGIMACHI K. (1989). Histologic features of minute and small human gastric adenocarcinoma. *Arch. Pathol. Lab. Med.*, **113**, 926–931.

NAGAYO T. (1975). Microscopical cancer of the stomach: a study of histogenesis of gastric carcinoma. *Int. J. Cancer*, **16**, 52–60.

SEKONS DH, MCSHERRY CK AND CALHOUN WF. (1984). Contribution of endoscopy to diagnosis and treatment of carcinoma of the stomach. *Am. J. Surg.*, **147**, 662–665.

SUGANO H, NAKAMURA K AND KATO Y. (1982). Pathological studies of human gastric cancer. *Acta Pathol. Jpn* (Suppl. 2), 329–347.

TSO PL, BRINGAZE WL, DAUTERRIVE AH, CORREA P AND COHN JR I. (1987). Gastric carcinoma in young. *Cancer*, **59**, 1362–1366.

XUAN XZ, UEYAMA T, YAO T AND TSUNEYOSHI M. (1993). Time trends of early gastric carcinoma. *Cancer*, **72**, 2889–2894.